# Identifying an immunogenic cell death-related gene signature and HSPA6 infers adverse prognosis in acute myeloid leukemia

**DOI:** 10.7150/jca.115717

**Published:** 2025-07-28

**Authors:** Ke Min, Chuanyang Lu, Xiaolu Wu, Shiyang Zhong, Ping Ma, Yu Zheng, Kai Xue, Qian Sun, Wenjie Liu, Chunling Wang, Liang Yu, Kening Li, Ming Hong, Ran Li

**Affiliations:** 1 Department of Hematology, the First Affiliated Hospital with Nanjing Medical University, Nanjing, China.; 2 Department of Hematology, The Affiliated Huaian No.1 People's Hospital of Nanjing Medical University, Northern Jiangsu Institute of Clinical Medicine, Huaian, China; 3 Department of Child Health Care, Women's Hospital of Nanjing Medical University, Nanjing Maternity and Child Health Care Hospital, Nanjing, China.; 4 Department of Bioinformatics, Nanjing Medical University, Nanjing, China.; 5 Collaborative Innovation Center for Personalized Cancer Medicine, Jiangsu Key Lab of Cancer Biomarkers, Prevention and Treatment, Nanjing Medical University, Nanjing, China.; 6 Shanghai Institute of Hematology, State Key Laboratory of Medical Genomics, National Research Center for Translational Medicine at Shanghai, Ruijin Hospital, Shanghai Jiao Tong University School of Medicine, Shanghai, China.; 7 Biomedical Big Data Center, Nanjing Medical University, Nanjing, China.

## Abstract

Immunogenic cell death (ICD) represents a specific form of regulatory cell death that initiates an adaptive immune response. We aimed to investigate the significance of immunogenic cell death-related genes (ICDGs) in AML, utilizing a combination of bioinformatics analysis, consensus clustering, functional enrichment analysis, and experimental validation in cell lines and animal models. Here, we identified 34 ICDGs, and single-cell analysis indicated that CD8^+^ T cells and NK cells exhibited gene expression patterns closely associated with ICD. Consensus clustering revealed two distinct subtypes of AML (ICD-high and ICD-low), with the former correlating with more favorable clinical outcomes and heightened infiltration of immune cells. A predictive model was established through LASSO regression, yielding a risk signature comprising six key ICDGs (FGFBP2, GZMB, ALPK2, NELL2, OPTN, FCGR2B), which successfully categorizes patients into high-risk and low-risk cohorts based on overall survival outcomes. Notably, HSPA6 emerged as a critical ICDG, with its knockdown in OCI-AML3 cells significantly inhibiting proliferation and inducing apoptosis, suggesting its potential as a therapeutic target. In summary, our research emphasizes the importance of ICD-related genes in predicting the prognosis of AML and initiates the development of a prognostic risk signature that may pave the way for personalized treatment strategies while highlighting the need for further validation and exploration of HSPA6 in AML.

## Introduction

Acute myeloid leukemia (AML) represents a heterogeneous hematological malignancy distinguished by the clonal expansion of myeloid progenitor cells, resulting in impaired hematopoiesis and significant morbidity and mortality among affected individuals^1^. The complexity of AML is underscored by its diverse genetic and epigenetic alterations, which contribute to its variable clinical presentation and response to treatment. Despite advancements in therapeutic strategies, including chemotherapy and targeted therapies, the prognosis for many AML patients remains poor^2^, primarily due to high rates of relapse and treatment resistance, highlighting the urgent need for novel prognostic markers and therapeutic targets to improve patient outcomes.

Immunogenic cell death (ICD) represents a distinctive form of cellular demise characterized by the exposure of a “find me” signal, which is revealed through the liberation of tumor-associated antigens (TAAs) and tumor-specific antigens (TSAs), thereby initiating an adaptive immune response^3^. The essence of the ICD phenomenon lies in the release of damage-associated molecular patterns (DAMPs) from dying tumor cells, which subsequently incites the activation of immune responses specific to the tumor^4^. DAMPs consist of the exposure of calreticulin (CRT) on the cell surface, the extracellular release of adenosine triphosphate (ATP), heat-shock proteins (HSP70 and HSP90), type I interferons (IFNs), high-mobility group box-1 (HMGB1), and various members of the interleukin-1 (IL-1) cytokine family^5^. ICD is triggered by specific chemotherapeutic agents, oncolytic viruses, physicochemical modalities, photodynamic techniques, and radiation therapy; the long-term effectiveness of anti-cancer medications can be attributed to the activation of adaptive immune responses^6^. As an important process of anti-tumor immunotherapy, additional investigations are necessary to clarify the mechanisms underlying the emergence of ICD for effective tumor treatment.

Previous research categorized patients into two subtypes related to ICD by utilizing ICDGs and formulated a prognostic signature associated with ICD based on the differentially expressed genes (DEGs) between these two subtypes^7^, ^8^. Both studies included the TCGA-LAML cohort and failed to exclude acute promyelocytic leukemia (APL, M3) patients. APL is the only one that has been cured through treatment regimens combining retinoic acid and arsenic trioxide^9^. Hence, the prognosis-related conclusions based on mixed AML patients including M3 patients are not reliable. Also, we explored the biological role of HSPA6 in AML using wet lab validation in cell lines and animal models.

In this study, non-M3 patients from the TCGA-LAML cohort were divided into two ICD-related subtypes, and the survival and tumor microenvironment differences between subtypes were investigated. An ICD-related prognostic signature was established to predict the prognosis in patients with AML. In addition, HSPA6 promoted AML growth in vitro and in vivo. The outcomes from this study are expected to make a substantial contribution to the comprehension of the function of ICDGs in AML.

## Materials and Methods

**Figure 1:**
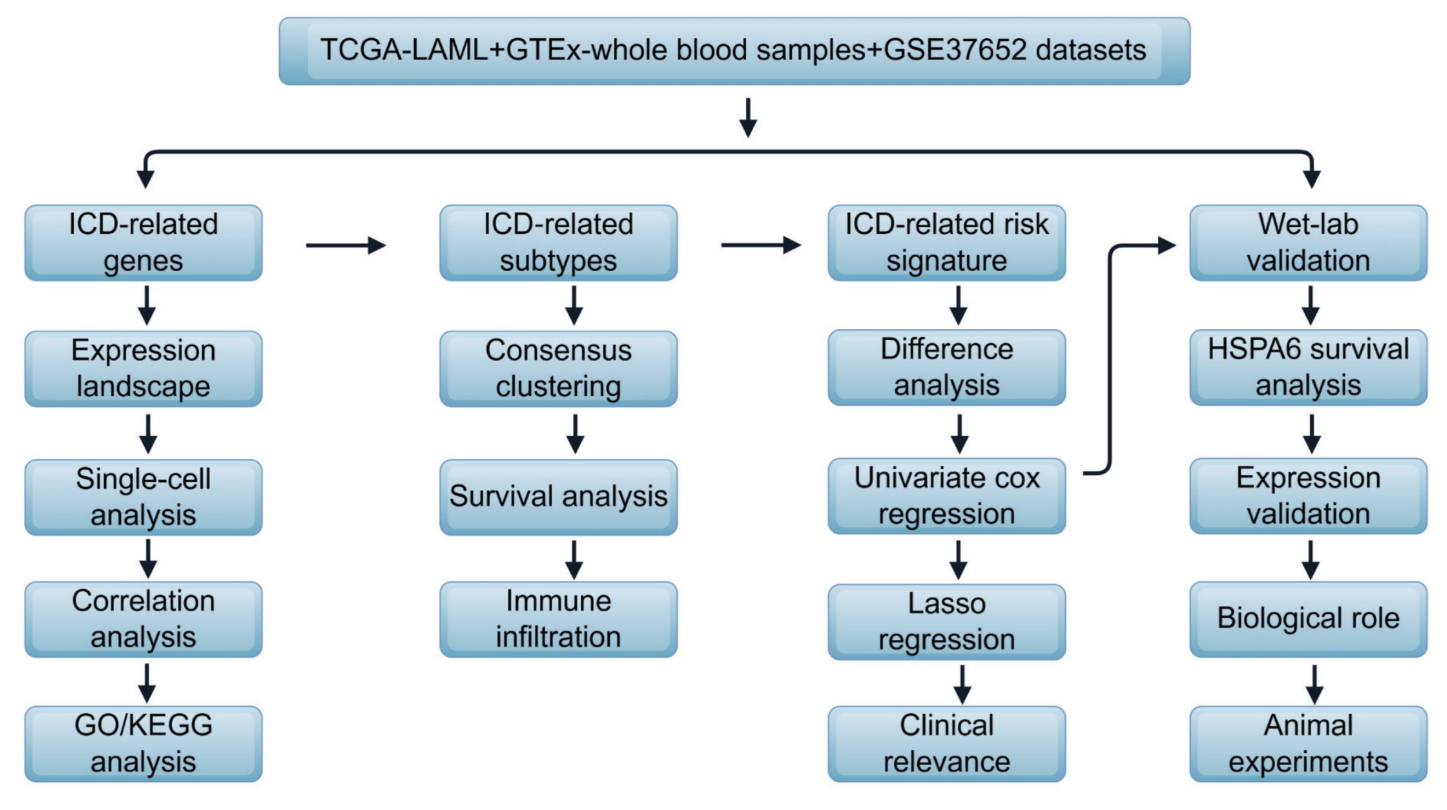
Flow diagram of the study, drawn by Figdraw.

The conceptual diagram of the current study is shown in **Figure 1**. The detailed methods are shown in the supplementary materials.

### Data acquisition

All eligible samples were gathered in accordance with the following inclusive criteria: (1) specimens diagnosed with AML; (2) existence of transcriptome data; (3) provision of general survival statistics and pertinent clinical details. Patients diagnosed with acute promyelocytic leukemia (M3) were excluded from this analysis. A total of 103 AML patients from the TCGA database were designated as the training cohort. Additionally, 444 GTEx-whole blood samples served as normal controls. Furthermore, 422 AML patients from the GSE37642_GPL96 cohort within the Gene Expression Omnibus (GEO) database were chosen as the validation cohort.

Clinical and transcriptomic data for both the TCGA-LAML and GTEx whole blood cohorts were procured from the UCSC Xena database (http://xena.ucsc.edu/). All transcriptome datasets underwent normalization as delineated by the Xena database at the University of California, San Diego. Microarray data for the validation cohort was acquired from the Gene Expression Omnibus (GEO). Probes were converted into gene symbols based on the corresponding probe mappings of the platform. In cases where a single probe corresponded to multiple genes, the probe was eliminated. Conversely, if multiple probes mapped to the same symbol, the median expression level was utilized.

Single-cell RNA sequencing (scRNA-seq) data generated from 40 newly diagnosed AML bone marrow samples were integrated from four studies^10^-^13^. A total of 272,946 cells were included for subsequent analysis.

### Statistical analysis

Statistical evaluations were conducted utilizing R software, version 4.1.2. The Wilcoxon test served to assess the disparities between the two cohorts, while the Kruskal-Wallis test was employed to examine variances among several groups. A p-value of less than 0.05 was deemed statistically significant (*P < 0.05, **P < 0.01, ***P < 0.001).

## Results

### Biological function analysis of ICD-related genes

**Figure 2:**
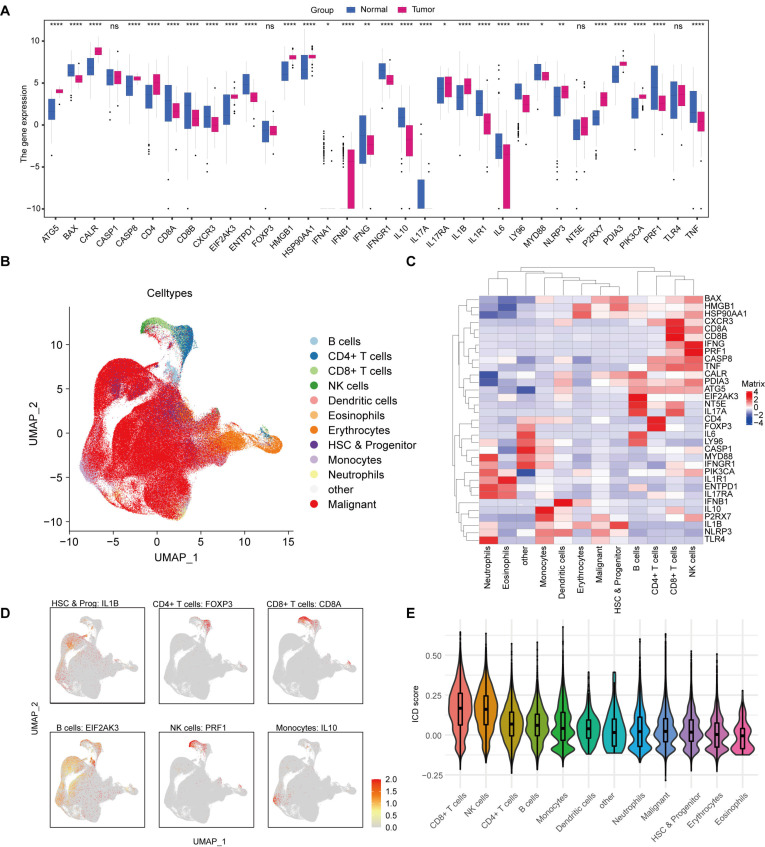
**Genetic characteristics of ICDGs in AML.** (A) The expression of ICDGs among TCGA-LAML and GTEX-whole blood normal controls. (B) t-SNE plot of the bone marrow cell landscape colored by cluster. (C) Heatmap of the expression levels of ICDGs among 12 subclusters of bone marrow cells. (D) Featureplots showing the normalized expression of representative ICDGs in each subcluster. (E) Violin plots showing the ICD score among 12 subclusters.

First, we examined the expression profiles of 34 ICD-related genes (ICDGs) in both normal samples and those from patients with acute myeloid leukemia (AML) sourced from the TCGA-LAML and GTEx databases. The findings revealed that 14 ICDGs exhibited up-regulation, while 16 showed down-regulation, with 4 ICDGs displaying no significant differences (Figure 2A). To further gain insights into the distribution of ICDGs in immune cells, we integrated bone marrow scRNA-seq data from 40 newly diagnosed AML patients. Totally 272,946 cells were integrated after quality control. We performed unsupervised clustering of all these cells and identified 12 transcriptional cell clusters (Figure 2B). Heatmaps and featureplots were generated to visualize ICDGs of each cluster of bone marrow cells (Figure 2C-2D). We then scored 12 clusters according to the expression of ICDGs (Figure 2E). The results showed that multiple cell types exhibited gene expression patterns closely associated with immunogenic cell death, especially CD8^+^ T cells and NK cells.

To explore the interrelations among these ICDGs, we conducted a protein-protein interaction (PPI) network analysis (Figure S1A). In order to further elucidate the connections between the 34 ICDGs, a correlation analysis was performed within the TCGA-LAML cohort (Figure S1B). The results represented in Figures 2B and 2C indicated that the 34 ICDGs demonstrated statistically significant correlations amongst one another. Gene Ontology (GO) and KEGG pathway analyses revealed that several gene sets were linked to the 34 ICDGs within the TCGA-LAML cohort. The GO enrichment analysis highlighted that ICDGs were predominantly involved in the positive regulation of cytokine production and T-cell activation processes. KEGG analysis indicated a robust association of these genes with lipid metabolism, atherosclerosis, and influenza A (Figure S1C).

### Consensus clustering unveiled ICD‑related subtypes

**Figure 3:**
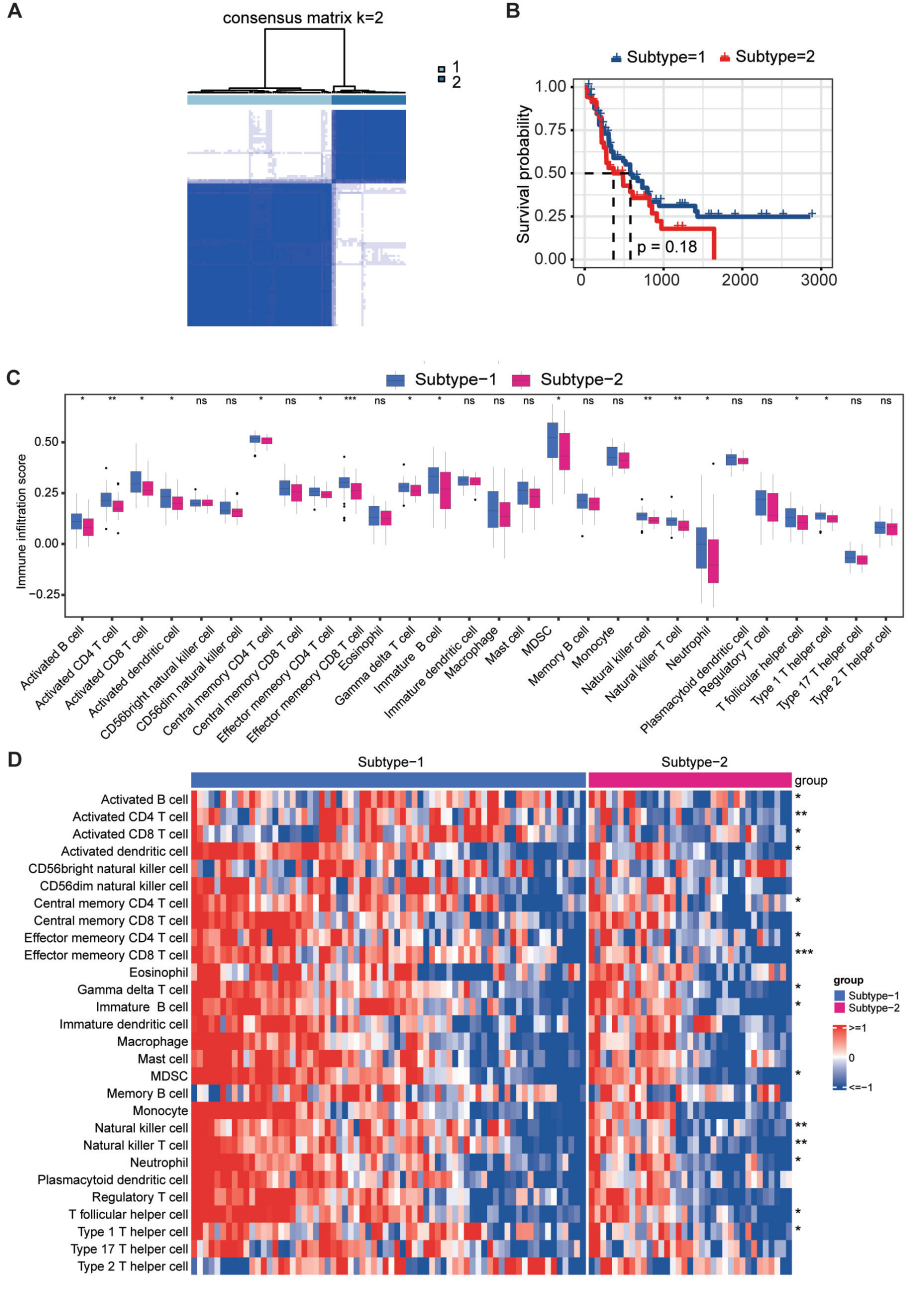
** Identification of ICD-related molecular subtypes.** (A) Two ICD-related subtypes were identified by consensus clustering. (B) KM curve analysis for OS between subtypes. (C-D) Differences in immune infiltration analysis between subtypes.

Consensus clustering analysis was conducted on TCGA-LAML to identify clusters of AML associated with ICD. Following k-means clustering, two distinct clusters were revealed, each characterized by unique ICDG expression profiles (Figure 3A). Overall, subtype-1 exhibited a pronounced ICDG pattern, while subtype-2 demonstrated diminished levels of ICDG expression (Figure S1). Therefore, we defined subtype-1 as the ICD-high subtype and subtype-2 as the ICD-low subtype. The ICD-high subtype was associated with favorable clinical outcomes and the ICD-low subtype presented a poor prognosis (Figure 3B). We next explored the infiltration of different immune cell types between two subtypes. A wealth of anti-tumor lymphocyte subsets was observed infiltrating the ICD-high subtype, including activated B cells, activated CD4+ T cells, activated CD8+ T cells, central memory CD4+ T cells, and effector CD8+ T cells (Figure 3C-3D). These findings suggested a connection between the ICD-high subtype and an immune-activating phenotype, while the ICD-low subtype appeared to be associated with an immunosuppressive phenotype. A differential expression analysis was performed between the two subtypes, leading to the identification of 377 ICD-related genes. The outcomes of the gene enrichment analysis revealed that immune-related biological processes played a pivotal role (Figure S2).

### Development and validation of the ICD risk signature

**Figure 4:**
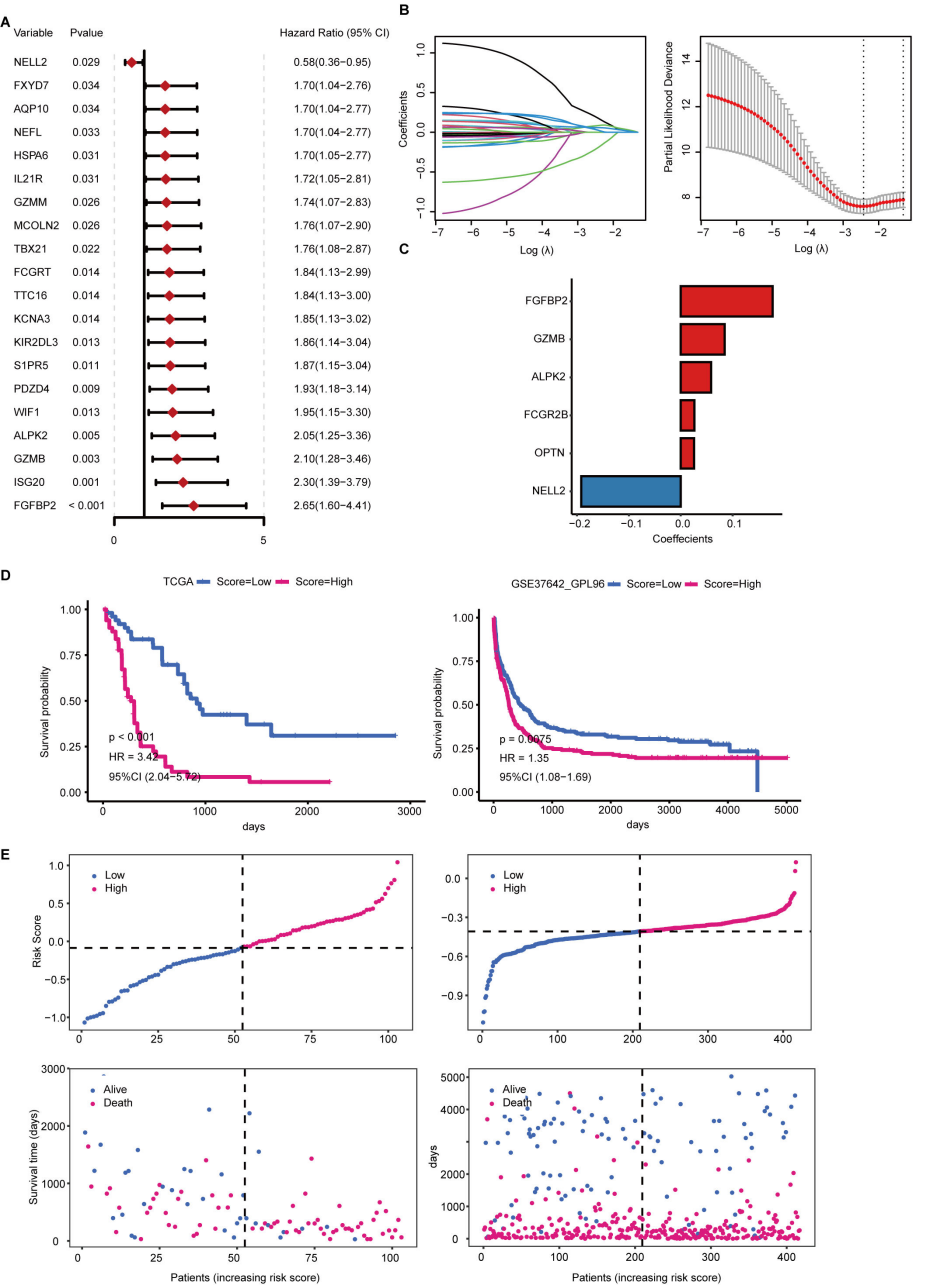
** Construction of an ICDG prognostic model.** (A) Forest plot for the top 20 DEGs using the univariate Cox regression analysis. (B) LASSO regression was performed to calculate the minimum criteria. (C) The coefficients of six genes were estimated by multivariate Cox regression. (D) Kaplan-Meier analysis for the risk groups in training and validation sets. (E) The distribution of risk scores and survival status of AML patients in training and validation sets.

We conducted a univariate Cox regression analysis to uncover ICDGs associated with overall survival (OS), identifying a total of 28 genes deemed prognostic ICDGs. The top 20 ICDGs were shown in the form of a forest plot (Figure 4A). Six genes (FGFBP2, GZMB, ALPK2, NELL2, OPTN, and FCGR2B) were filtered out to create an ICD risk signature using LASSO regression (Figure 4B). The corresponding coefficients were generated to calculate the risk score (Figure 4C).

To assess the efficacy of the ICD risk signature in forecasting prognosis, we computed the risk score for both the training cohort (TCGA-LAML) and the validation cohort (GSE37642_GPL96), subsequently categorizing patients into distinct risk categories according to the median risk score. Kaplan-Meier survival curve analysis revealed that individuals with low-risk scores exhibited markedly superior overall survival (OS) compared to those with high-risk scores across both the training and validation cohorts (Figure 4D). The risk curve illustrated that an increase in risk scores corresponded with a diminishing survival duration for AML patients, alongside a rise in mortality rates (Figure 4E). These findings suggest that the ICD risk signature provides a robust assessment of the prognosis for AML patients.

**Figure 5:**
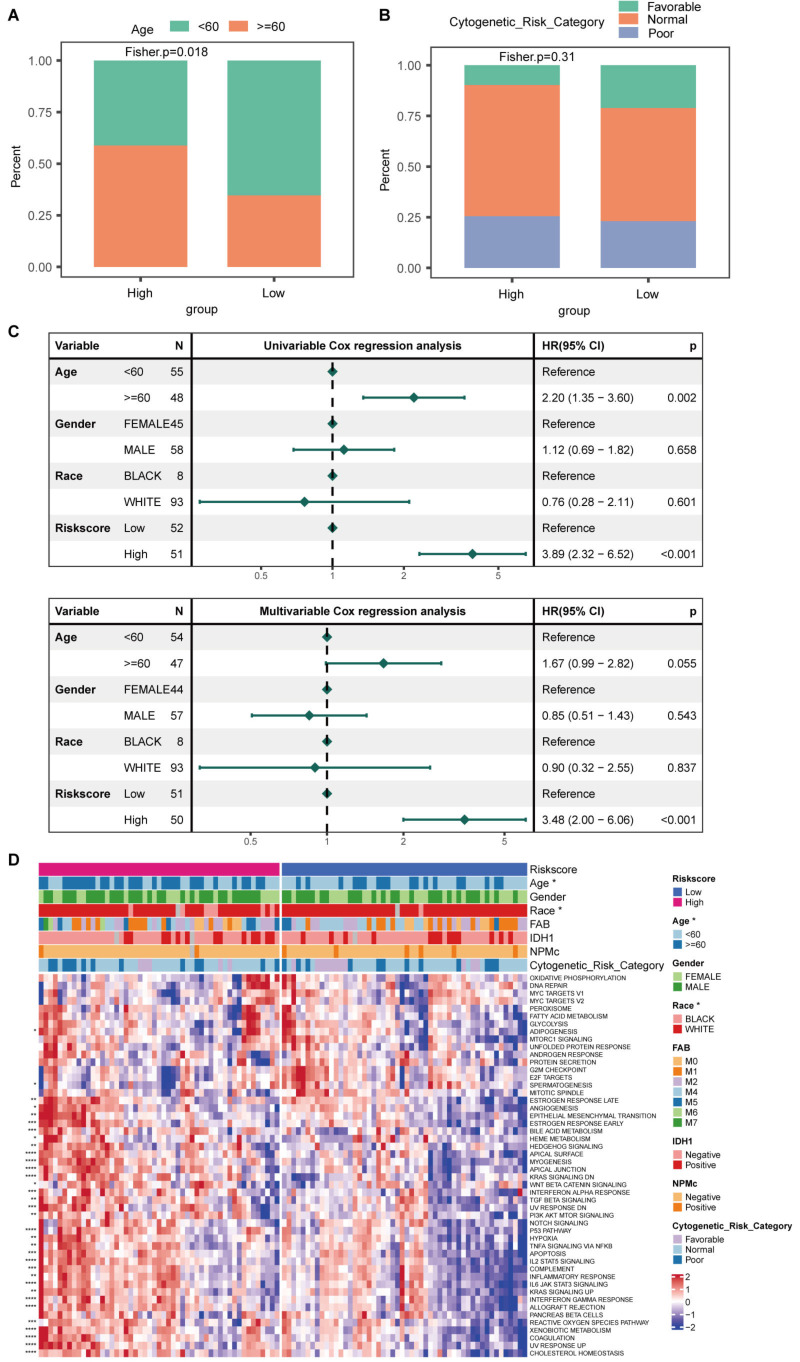
** The relationship between risk score and clinical features.** Age (A) and cytogenetic risk (B) composition of patients within the high- and low-risk groups. (C)Univariate and multivariate analyses for clinical features and risk score. (D) Pathway enrichment analysis for different groupings based on clinical features and risk score.

Next, we asked whether the distribution of risk score was associated with clinicopathological characteristics of AML patients. We found the majority of the high-risk group was over 60 years of age (Figure 5A). Regarding cytogenetic risk, the high-risk group had fewer patients with favorable cytogenetic risk (Figure 5B). We performed univariate and multivariate Cox analyses and found that risk score was an independent prognostic factor (Figure 5C). Pathway enrichment analysis was conducted between the two risk categories, revealing that the notable signaling pathways of IL2-STAT5, IL6-JAK-STAT3, TNF-alpha, NF-κB, and KRAS were predominantly enriched in the high-risk cohort (Figure 5D).

### Biological role of HSPA6 in the OCI-AML3 cell line

We next wanted to determine the role of genes from the ICD risk signature in AML cells. Firstly, we compared their expression between human AML cell lines and the whole blood samples from the CCLE database and the GTEx database, respectively. The results showed that FGFBP2, GZMB, ALPK2, and NELL2 expression is extremely low in AML cell lines (Figure S4). Also, the protein encoded by FCGR2B is a receptor for the Fc region of immunoglobulin gamma complexes. In addition, OPTN has been reported to be rate-limiting for AML cell proliferation^14^. Overall, the six genes from the ICD risk signature are either expressed in the non-tumor cells or has been reported in AML. Hence, we focused on the top 20 genes considered to be prognostic ICDGs in Figure 4A, and HSPA6 attracted our attention. HSPA6 is a heat shock protein that plays a crucial role in the cellular stress response and is intricately linked to the onset, progression, and prognosis of multiple cancers^15^. However, its role in AML is not clear.

**Figure 6:**
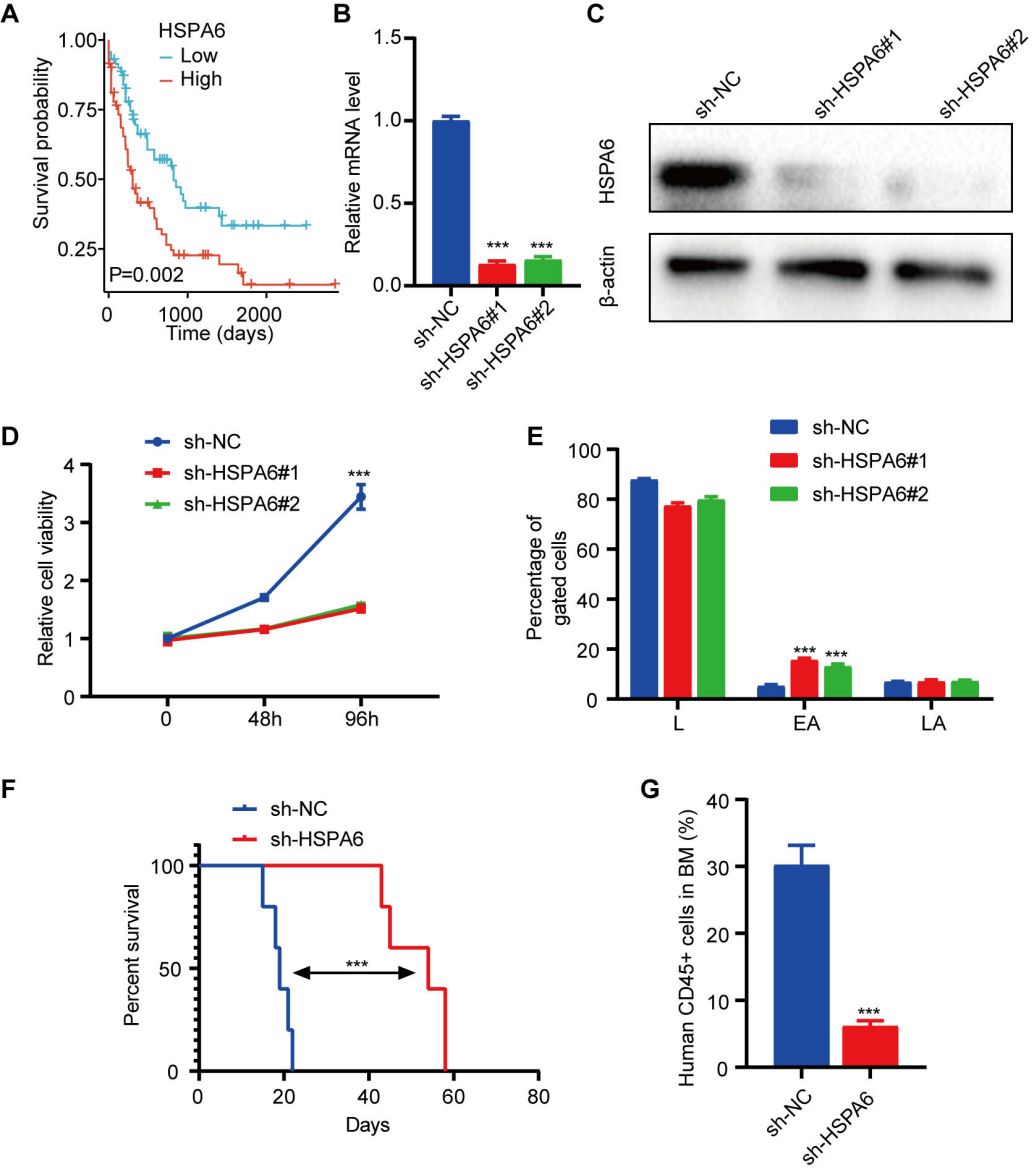
** HSPA6 promotes the growth of human AML cells.** (A) Kaplan-Meier analysis for patients divided into high- and low-HSPA6 expression groups in the TCGA-LAML cohort. The mRNA (B) and protein (C) levels of HSPA6 were assessed in OCI-AML3 cells transduced with sh-NC or sh-HSPA6. (D) Proliferation assays in OCI-AML3 cells. (E) The proportion of apoptotic cells in OCI-AML3 cells with sh-NC or sh-HSPA6. L: living cells; EA: early apoptosis; LA: late apoptosis. (F) Kaplan-Meier survival plots of NOD mice injected with OCI-AML3 cells transduced with sh-NC or sh-HSPA6. (G) The proportions of CD45+ cells in recipient mice.

Initially, we observed that patients exhibiting elevated levels of HSPA6 are associated with unfavorable outcomes (Figure 6A). Subsequently, the HSPA6 knockdown cellular model was established within the OCI-AML3 (Figure 6B-6C) and THP-1 (Figure S5A-S5B) cell lines. Loss-of-function investigations were conducted in the OCI-AML3 cell lines, revealing that the knockdown of HSPA6 led to considerable suppression of cell proliferation and marked induction of early apoptosis in the OCI-AML3 cell line (Figure 6D-6E). The results in the THP1 cell line were the same with the OCI-AML3 cell line (Figure S5C-S5D). HSPA6 knockdown notably prolonged the lifespan of mice receiving xenografts (Figure 6F). Additionally, HSPA6 knockdown resulted in a reduction of CD45+ cells in the bone marrow (Figure 6G).

## Discussion

Immunotherapy faces challenges due to the low immunogenicity of acute myeloid leukemia (AML), the presence of a tumor-immunosuppressive microenvironment, and mechanisms of immune evasion. The induction of immunogenic cell death (ICD) has surfaced as a promising approach to dismantle the tumor's immunosuppressive environment, stimulate a T-cell-mediated adaptive immune response, and ultimately elicit anti-tumor immunity for prolonged tumor control^16^, ^17^. Therefore, there may be an advantage in exploring the role of ICDGs in AML.

According to ICDG expression, consensus clustering analysis was used to classify AML patients into two ICD subtypes. The two subtypes differed significantly in terms of immune infiltration and prognosis. Subtype-1 displayed a high ICDG pattern and infiltration of antitumor lymphocyte subsets ensued, which was responsible for the better prognosis of patients in subtype-1.

Various antitumor treatments stimulate immunogenic cell death (ICD), leading to the release of danger-associated molecular patterns (DAMPs) and tumor-associated antigens, which facilitate the maturation of dendritic cells (DCs) and the infiltration of cytotoxic T lymphocytes into tumors. This process can effectively counteract the immunosuppressive tumor microenvironment, thereby enhancing the efficacy of immunotherapy^17^. ICD can release antigens and DAMPs, such as calreticulin (CRT), adenosine-5′-triphosphate (ATP), and heat shock proteins (HSPs). When it binds to CD91, CRT acts as an "eat-me" signal, facilitating the recognition of tumor-associated antigens (TAAs) by antigen-presenting cells (APCs)^18^. Dying cells trigger autophagy, resulting in the secretion of ATP that attaches to P2X7 receptors on phagocytes, acting as a "find-me" signal to summon macrophages and dendritic cells^19^. In addition, HSPs have the ability to draw in phagocytes and stimulate the activation of natural killer (NK) cells^20^. In general, the liberated DAMPs have the capacity to attract antigen-presenting cells (APCs) and promote the maturation of dendritic cells (DCs), the activation of cytotoxic T lymphocytes (CTLs), and the release of various cytokines linked to both innate and adaptive immune responses, thereby eliciting robust immune reactions. The accumulation of antitumor T cells and cytokines alters the tumor microenvironment from a "cold" immunosuppressive state to a "hot" immune-responsive condition, ultimately leading to improved prognoses^21^. In our study, the ICD-high subtype enriched with more antitumor lymphocyte subsets had a better prognosis.

Based on the DEGs between two subtypes, we constructed an ICD-related signature for AML prognosis. Six key genes were involved in this signature. Fibroblast growth factor binding protein type 2 (FGFBP2) encodes a member of the fibroblast growth factor binding protein family, which is involved in cytotoxic lymphocyte-mediated immunity^22^. Granzyme B (GZMB) is a serine protease and the main regulator of cytotoxicity produced by lymphocytes. Four different granzymes (GZMA, GZMB, GZMH, and GZMM) were identified as elevated proteins linked to relapse in adult AML patients^23^. Alpha protein kinase 2 (ALPK2) is recognized as a pivotal player in oncogenesis through its regulation of cell cycle progression and the genes involved in DNA repair mechanisms^24^. Neural epidermal growth factor-like 2 (NELL2) is an extracellular glycoprotein primarily found in the nervous system. Acting as a downstream target of E2F1, the transcriptional activation of NELL2 enhances the survival, migration, and invasion of NSCLC cells^25^. Optineurin (OPTN), a well-recognized autophagy receptor, has received considerable attention due to its multifaceted roles in the autophagic process. Mitophagy selectively destroys mitochondria to maintain mitochondrial homeostasis, which in turn maintains and progresses AML disease. Fc Gamma Receptor IIb (FCGR2B), which encodes FcγRIIB, serves as a low-affinity receptor for the Fc segment of immunoglobulin gamma complexes. It plays a crucial role in the phagocytosis of immune complexes and the modulation of antibody production by B-cells. A significant portion of the six genes is linked to the tumor immune microenvironment, offering insights and guidance for our forthcoming investigations.

HSPA6 (Heat Shock Protein Family A Member 6) is a molecule that belongs to the HSP70 family and is situated on human chromosome 1q23.3. It encodes a protein that comprises two crucial domains: the N-terminal nucleotide-binding domain and the C-terminal substrate-binding domain. HSPA6 plays a pivotal role in various cancers, facilitating tumor initiation and progression while simultaneously suppressing certain tumor types^15^. As a constituent of the heat shock protein (HSP) family, HSPA6 is involved in cellular stress responses, protecting cells from damage under various stress conditions. In the cancer microenvironment, the expression of HSPA6 is significantly upregulated, which is closely related to tumor cell survival, proliferation, and chemoresistance^26^. In terms of cellular signaling pathways, HSPA6 is closely associated with multiple key regulatory networks. For instance, HSPA6 can modulate the ERK/MAPK signaling pathway, affecting the transmission of cell proliferation and survival signals^27^. Additionally, HSPA6 interacts with transcription factors such as p53 and NF-κB, regulating the expression of downstream genes and thereby participating in tumor initiation and development^28^. The complexity of these interactions underscores the significance of HSPA6 in various biological processes of cancer. Additionally, our findings regarding the biological role of HSPA6 provide insights into the mechanisms underlying AML cell survival and proliferation. The role of HSPA6 in facilitating cell survival positions it as a potential therapeutic target. Further exploration of HSPA6's interaction with molecular pathways may yield novel strategies for enhancing treatment efficacy in AML.

In conclusion, this study provides evidence for the significant role of ICDGs in the prognosis of acute myeloid leukemia. The insights gained from our analysis, particularly regarding the biological role of key ICDGs such as HSPA6, may inform the development of novel therapeutic targets aimed at improving patient prognosis in AML.

## Supplementary Material

Supplementary figures and methods.
