# Supplementary material for: Identifying an immunogenic cell death-related gene signature and HSPA6 infers adverse prognosis in acute myeloid leukemia
Source: J Cancer. 2025 Jul 28;16(12):3643–53. doi: 10.7150/jca.115717 (PMC12435325; doi:10.7150/jca.115717)
Supplement: Supplementary file 1 — Supplementary figures and methods. [file jcav16p3643s1.pdf]

Supplementary materials and methods

Analysis of single-cell seq data

In order to reduce the impact of batch effects on the analyzed results, the harmony method was used to process the data for batch correction and integration[1]. It eliminates unnecessary batch/technique differences when integrating multiple single-cell datasets, allowing cells of the same type to be clustered together rather than artificially separated due to differences in experimental conditions. Subsequently, the integrated single-cell data are downscaled and visualized using the uniform manifold approximation and projection (UMAP) algorithm. Cell type annotation was performed using the "singleR" package of R software[2]. We loaded the Blueprint Encode Data and the Novershtern Hematopoietic Data as the reference dataset and used the singleR algorithm to define cell subsets. The IFNA1 gene from 34 ICDGs was not found in the integrated scRNA-seq. The average expression heatmap of 33 ICDGs in different cell types was then plotted, and Featureplot was used to show the spatial distribution of expression of representative ICDGs genes. The AddModuleScore was used to score the set of ICDGs genes in order to assess the activity level of gene expression patterns closely associated with immunogenic death in different cells. Gene set scores were calculated for each cell using the "Seurat" R package. Ultimately, each cell receives a corresponding score that can be used for further analysis.

Consensus clustering analysis

The 34 ICD genes were extracted from Table S1 featured in an extensive meta-analysis[3]. The ConcensusClusterPlus tool in R was employed to conduct consensus clustering, aiming to pinpoint molecular subtypes linked to ICD. Ideal cluster numbers between k = 2–10 were assessed, and this process was replicated 1,000 times to guarantee stable results. The heat map tool in R was utilized to draw a cluster map.

Functional enrichment analysis

The "limma" R package was employed to identify differentially expressed genes (DEGs) among subtypes or risk categories. The criteria for significance were established as $|logFC| > 1$ and an adjusted p-value < 0.05. Functional analysis through Gene Ontology (GO) and pathway analysis via the Kyoto Encyclopedia of Genes and Genomes (KEGG) were conducted using the "ClusterProfiler" R package. Gene set enrichment analysis (GSEA) was executed with GSEA software.

Network analysis

A network provides a powerful approach for depicting protein-protein interactions (PPI). The Search Tool for the Retrieval of Interacting Genes (STRING) database was utilized to forecast the PPI network of the 34 ICDGs, offering us valuable insights into the intricate dynamics of protein interactions. A PPI network was assembled using Cytoscape software (NIH, National Resource for Network Biology).

Development and evaluation of the ICDG signature

The “limma” R package was employed to identify differentially expressed genes (DEGs) across subtypes or risk categories. The significance thresholds established were $|logFC| > 1$ and an adjusted p-value < 0.05. A total of 377 ICD-related differentially expressed genes emerged from the consensus clustering subtypes. A univariate Cox regression analysis was conducted to assess the correlation between the expression levels of individual ICD-related genes and overall survival (OS). To mitigate the risk of overfitting, a LASSO regression analysis was performed to construct a prognostic model. The risk score was computed using the formula: $\beta1 \times$ gene1 expression + $\beta2 \times$

gene2 expression + … + βn × genen expression, where β represents the correlation coefficient. All patients with acute myeloid leukemia (AML) were categorized into high-risk and low-risk groups based on their median risk scores. The overall survival disparity between the high-risk and low-risk cohorts was evaluated using the Kaplan-Meier (KM) curve. Fisher's exact test was applied to compare the age distribution and cytogenetic risk levels between groups stratified by risk.

Cell culture

OCI-AML3 and THP1 cells were sourced from DSMZ and cultivated in Roswell Park Memorial Institute (RPMI-1640) medium (Sigma), enriched with 10% fetal bovine serum (FBS, Sigma), 1% antibiotic-antimycotic, and 1% L-glutamine. Cell line culture was performed in 37℃ /5% CO_2.

Xenograft investigations

The xenograft murine model was established by introducing 1×10^6 OCI-AML3 cells harboring sh-NC or sh-HSPA6 into NOD.Cg-Prkdc^scidIl2rg^tm1Wjl/SzJ (NSG) mice, aged between 6 to 8 weeks. Identification of human CD45+ cells within the bone marrow was conducted through flow cytometry. All animal studies received endorsement from the Institutional Animal Care and Use Committee at Nanjing Medical University.

Quantitative real-time PCR

Total RNA was isolated from cell lines using TRIzol reagent (Invitrogen, United States). Quantitative real-time PCR (qRT-PCR) was performed as previously described[4]. The primers employed for qRT-PCR were:

5′-CAAGGTGCGCGTATGCTAC-3′ (Forward Primer),

5′-GCTCATTGATGATCCGCAACAC-3′ (Reverse Primer).

Western blotting

Western blotting was performed as previously described [4]. The primary antibodies, HSPA6 and GAPDH, were obtained from Abcam, USA. Secondary antibodies, either anti-mouse or anti-rabbit, were acquired from Cell Signaling Technology, USA.

Transfection

The lentivirus containing HSPA6 knockdown or a negative control sequence (NC) was obtained from OBIO (Obio Technology Corp, China). The transduction process was performed on OCI-AML3 and THP1 cells. Stable pools of transductants were established through selection with puromycin (1 μg/ml) over a duration of two weeks.

Cell proliferation/growth and apoptosis assays

Cell proliferation was evaluated using the Cell Counting Kit-8 (CCK-8) proliferation assay (Dojindo, Japan) in accordance with the manufacturer’s guidelines. For apoptosis assessments, the Annexin V-FITC/PI cell apoptosis kit (Cat. No: KGA108, KeyGEN BioTECH) was utilized following the manufacturer’s instructions.

Supplementary table 1

Table S1. The list of 34 ICD genes

ENTPD1

NT5E

CALR

HMGB1

HSP90AA1

ATG5
BAX
CASP8
PDIA3
EIF2AK3
PIK3CA
CXCR3
IFNA1
IFNB1
IL10
IL6
TNF
CASP1
IL1R1
IL1B
NLRP3
P2RX7
LY96
MYD88
TLR4
CD4+
CD8+A
CD8+B
FOXP3
IFNG
IFNGR1
IL17A
IL17RA
PRF1

Supplementary figures

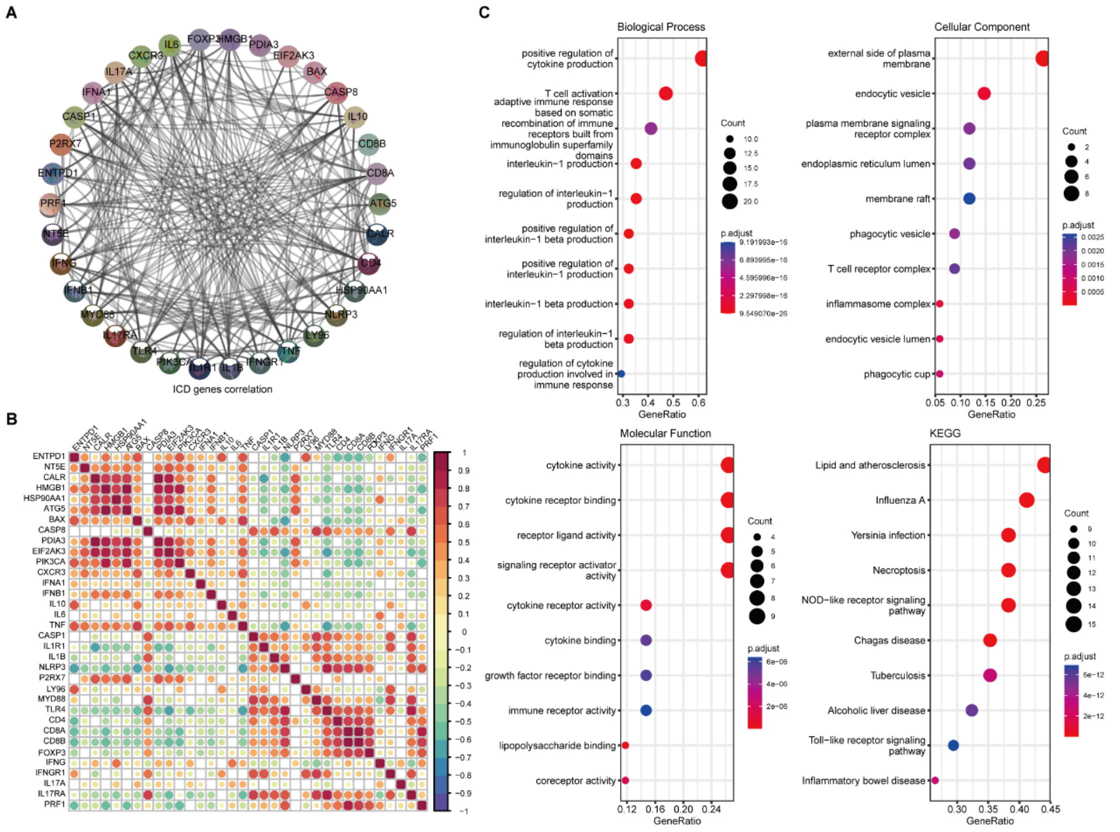

Figure S1. Biological function analysis of ICD-related genes (A) The PPI analysis for ICDGs in AML patients. (B) The correlation of ICDGs in AML patients. (C) GO and KEGG functional analysis of ICDGs.

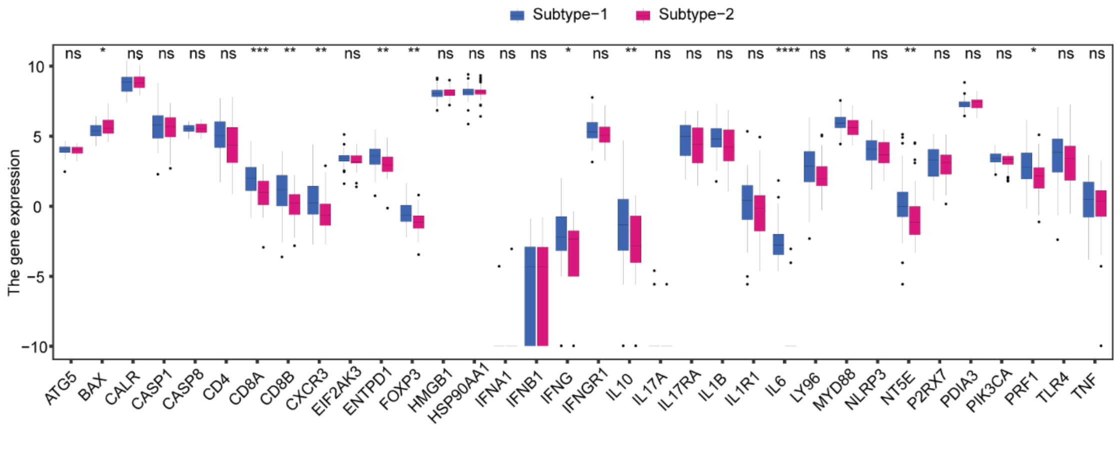

Figure S2. The expression of 34 ICDGs between two subtypes.

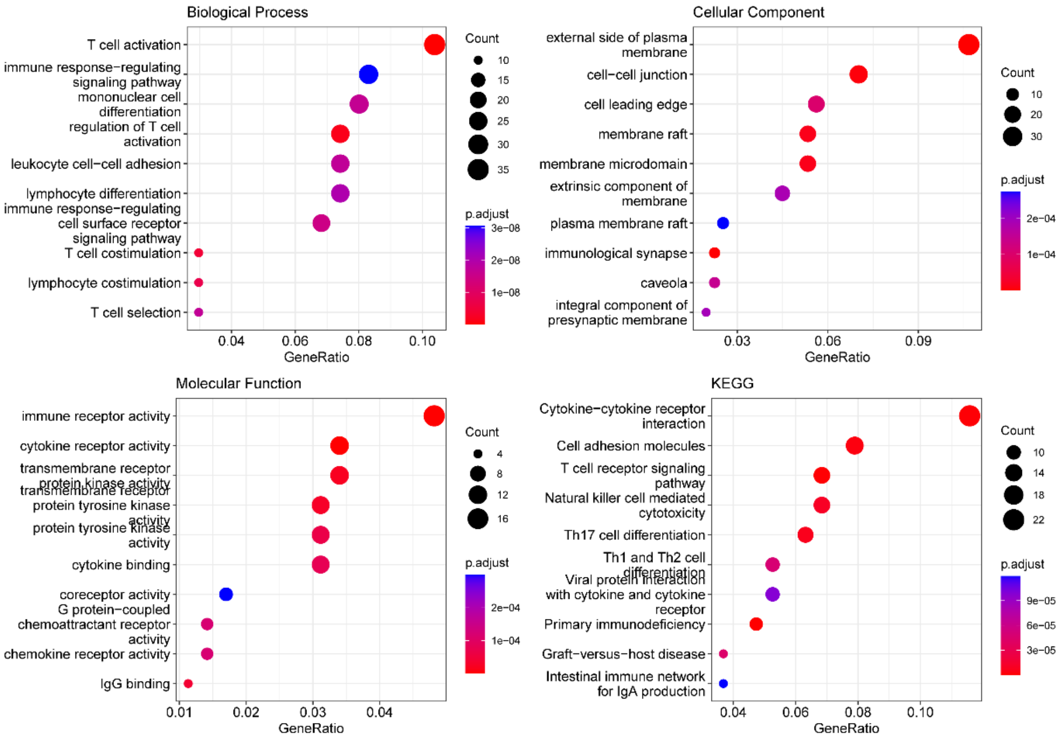

Figure S3. The functional enrichment analysis of 377 DEGs between risk groups.

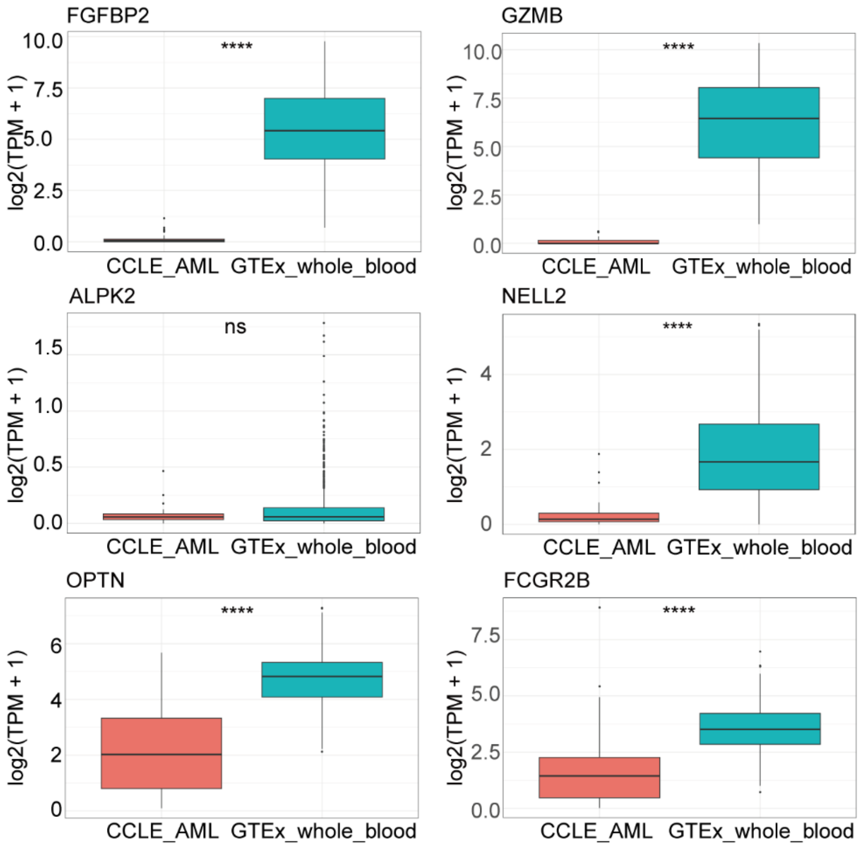

Figure S4. The expression of six genes from the risk signature between AML cell lines and the whole blood.

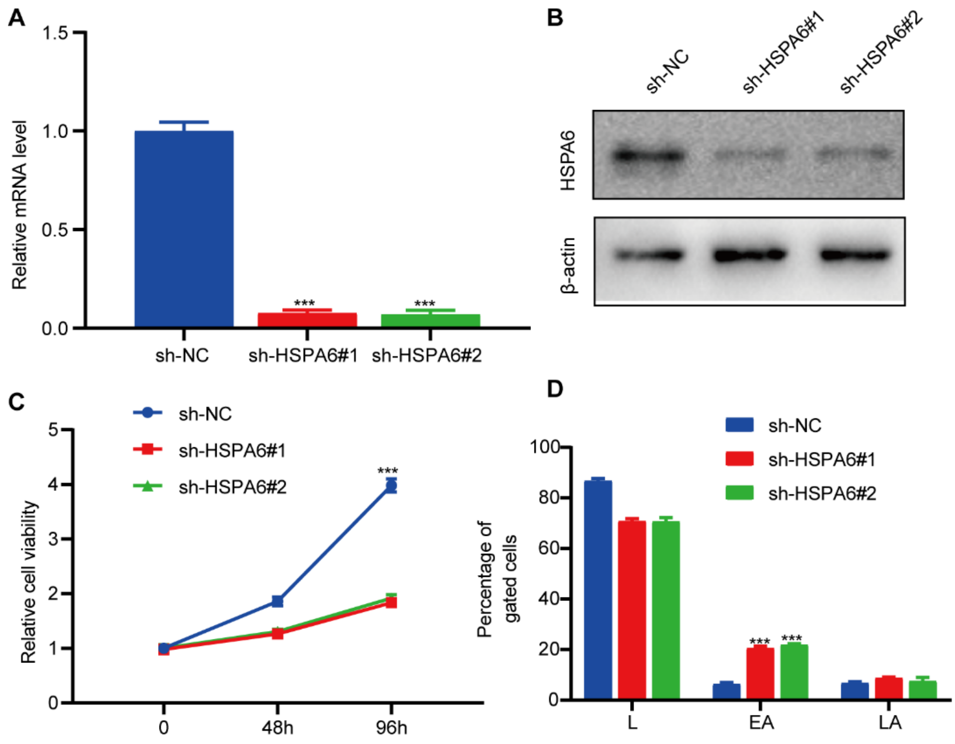

Figure S5. Biological role of HSPA6 in the THP-1 cell line. The mRNA (A) and protein (B) levels of HSPA6 were assessed in THP1 cells transduced with sh-NC or sh-HSPA6. (C) Proliferation assays in THP1 cells. (D) The proportion of apoptotic cells in THP1 cells with sh-NC or sh-HSPA6. L: living cells; EA: early apoptosis; LA: late apoptosis.

Reference:

1. Korsunsky I, Millard N, Fan J, Slowikowski K, Zhang F, Wei K, et al. Fast, sensitive and accurate integration of single-cell data with Harmony. Nat Methods. 2019; 16: 1289-96.

2. Aran D, Looney AP, Liu L, Wu E, Fong V, Hsu A, et al. Reference-based analysis of lung single-cell sequencing reveals a transitional profibrotic macrophage. Nature immunology. 2019; 20: 163-72.

3. Garg AD, De Ruysscher D, Agostinis P. Immunological metagene signatures derived from immunogenic cancer cell death associate with improved survival of patients with lung, breast or ovarian malignancies: A large-scale meta-analysis. Oncoimmunology. 2016; 5: e1069938.

4. Li R, Zhang L, Qin Z, Wei Y, Deng Z, Zhu C, et al. High LINC00536 expression promotes tumor progression and poor prognosis in bladder cancer. Exp Cell Res. 2019; 378: 32-40.
